# Datawarehouse-enabled quality control of atrial fibrillation detection in the stroke unit setting

**DOI:** 10.1016/j.heliyon.2023.e18432

**Published:** 2023-07-19

**Authors:** Mario E. Andina, Alexander Nelde, Christian H. Nolte, Jan F. Scheitz, Manuel C. Olma, Michael Krämer, Eckhard Meisel, Anne Bingel, Andreas Meisel, Franziska Scheibe, Matthias Endres, Ludwig Schlemm, Christian Meisel

**Affiliations:** a Department of Neurology, Charité – Universitätsmedizin Berlin, Berlin, Germany; b Center for Stroke Research Berlin, Berlin, Germany; c Berlin Institute of Health, Berlin, Germany; d German Center for Cardiovascular Research (DZHK), Partner Cite Berlin, Germany; e NeuroCure Cluster of Excellence, Charité – Universitätsmedizin Berlin, Berlin, Germany; f German Center for Neurodegenerative Diseases (DZNE), Partner Site Berlin, Germany; g Bernstein Center for Computational Neuroscience, Berlin, Germany; h NeuroCure Clinical Research Center, Berlin, Germany; i Institute of Neuroradiology, Charité - Universitätsmedizin Berlin, Corporate Member of Freie Universität Berlin and Humboldt-Universität zu Berlin, Berlin, Germany; j Praxisklinik HerzKreislauf, Dresden, Germany; k Department of Internal Medicine and Cardiology, German Heart Center Berlin, Berlin, Germany; l Department of Radiology, Charité – Universitätsmedizin Berlin, Berlin, Germany

**Keywords:** Clinical data warehouse, Atrial fibrillation, ECG monitoring, Automated detection, Stroke unit, Alarm fatigue, Quality control

## Abstract

**Objective:**

(1) To assess the accuracy of a standard operating procedure (SOP) regarding the utilization of atrial fibrillation (AF) alarms in everyday clinical practice, and (2) to evaluate the performance of automated continuous surveillance for atrial fibrillation (AF) in hospitalized acute stroke patients.

**Design:**

Retrospective cohort study.

**Setting:**

Two stroke units from two tertiary care hospitals in Berlin, Germany.

**Participants:**

We identified 635 patients with ischemic stroke diagnosis for the time period between 01. January and 30. September 2021 of which 176 patients had recorded AF alarms during monitoring. Of those, 115 patients were randomly selected for evaluation. After excluding 6 patients with hemorrhagic stroke in their records, 109 patients (mean age: 79.1 years, median NIHSS at admission: 6, 57% female) remained for analysis.

**Intervention:**

Using a clinical data warehouse for comprehensive data storage we retrospectively downloaded and visualized ECG data segments of 65 s duration around the automated AF alarms. We restricted the maximum number of ECG segments to ten per patient. Each ECG segment plot was uploaded into a REDCap database and categorized as either AF, non-AF or artifact by manual review. Atrial flutter was subsumed as AF. These classifications were then matched with 1) medical history and known diseases before stroke, 2) discharge diagnosis, and 3) recommended treatment plan in the medical history using electronic health records.

**Main outcome measures:**

The primary outcome was the proportion of previously unknown AF diagnoses correctly identified by the monitoring system but missed by the clinical team during hospitalization. Secondary outcomes included the proportion of patients in whom a diagnosis of AF would likely have led to anticoagulant therapy. We also evaluated the accuracy of the automated detection system in terms of its positive predictive value (PPV).

**Results:**

We evaluated a total of 717 ECG alarm segments from 109 patients. In 4 patients (3.7, 95% confidence interval [CI] 1.18–9.68%) physicians had missed AF despite at least one true positive alarm. All four patients did not receive long-term secondary prevention in form of anticoagulant therapy. 427 out of 717 alarms were rated true positives, resulting in a positive predictive value of 0.6 (CI 0.56–0.63) in this cohort.

**Conclusion:**

By connecting a data warehouse, electronic health records and a REDCap survey tool, we introduce a path to assess the monitoring quality of AF in acute stroke patients. We find that implemented standards of procedure to detect AF during stroke unit care are effective but leave room for improvement. Such data warehouse-based concepts may help to adjust internal processes or identify targets of further investigations.

## 1 Introduction

Medical data acquisition and storage capabilities have increased over the last years providing unprecedented opportunities for the development and implementation of automated diagnostic and monitoring capabilities in many clinical settings. With the rise of these computational diagnostics and their transfer into clinical routine the need for quality control mechanisms increases, including assessments of diagnostic accuracy and evaluations of the effectiveness by which diagnostics are implemented in clinical operating procedures. Quality control and optimization of operating procedures related to automated alarm systems are also crucial to limit alarm fatigue [1].

Atrial fibrillation (AF) constitutes the most common cardiac arrhythmia and is estimated to cause every fifth ischemic stroke [2]. Up to 24% of acute stroke patients are considered to have AF newly diagnosed during the whole workup including the ambulatory diagnostics [3,4]. Especially paroxysmal fibrillation is difficult to detect and is potentially a relevant cause in patients with cryptogenic strokes [5]. Oral anticoagulants (OAC) constitute highly potent agents for prevention of stroke in patients with AF, lowering the risk of stroke occurrence by two-thirds [[6], [7], [8]]. Current stroke guidelines recommend short-term ECG recording for AF monitoring after an ischemic stroke combined with consecutive continuous ECG monitoring for at least 72 h [[9], [10], [11], [12], [13]]. The implementation of these recommendations may differ between hospitals.

Evaluating the performance of automated AF alarms and the effectiveness of implemented operating procedures to utilize these alarms in stroke unit settings are thus essential measures to guarantee treatment quality with respect to the identified stroke etiology. However, the validity of alarms, i.e., one of the center pillars determining treatment decisions in stroke patients, is often not known or insufficiently validated. Clinical data warehouses which allow the large-scale acquisition and storage of patient data from health information systems, electronic health records and patient monitoring systems are increasingly becoming available in many clinical centers. These structures may provide novel opportunities for the evaluation, validation and refinement of alarms and their ensuing operating procedures. In this retrospective cohort study, we report results of a processing pipeline built on a data warehouse structure in the setting of two stroke units. Our goals were (1) to assess the accuracy of a standard operating procedure (SOP) regarding the utilization of AF alarms in everyday clinical practice, and (2) to evaluate the performance of automated continuous surveillance of AF occurrence in hospitalized acute stroke patients.

## 2 Methods

### 2.1 Dataset

We initially selected all patients diagnosed with ischemic stroke (ICD-10: I63.-) admitted to two stroke units from tertiary care hospitals in Berlin, Germany, in the period between January 2021 and September 2021. The two stroke units with 18 monitoring beds were equipped with the Data Warehouse Connect (DWC) system (Philips) for long-term storage of monitoring data. The Charité/BIH (Berlin Institute of Health) Health Data Lake (HDL), a Hadoop-based platform that allows storage of a multitude of clinical, epidemiological, laboratory, and monitoring data, was used for data integration and analysis. Data usage and retrospective analysis were approved by the Institutional Review Board of Charité – Universitätsmedizin Berlin. Fig. 2 provides an overview of the data and data flow relevant to this study. The ECG data were recorded with Philips MP30 and MP50 monitors and stored as raw data in the data lake. The Philips monitor has an internal AF detection algorithm based on measurements of the R–R irregularity, the PR interval variability, and the P-wave variability. The system outputs alarm onset and announce times, both of which were also stored in the data lake. Beyond ECG and related alarms, the data lake also included a comprehensive set of additional parameters from each patient, including laboratory values, clinical scores, and diagnoses. For this study, we collected information about patient characteristics (age, sex), the National Institutes of Stroke Scale (NIHSS) score at admission and at discharge, and the total duration of monitoring until discharge from the clinical data warehouse. In addition, we collected data from the discharge letters whether oral anticoagulation (OAC) was prescribed based on indications and contraindications from the stroke register database [14]. This information allowed us to assess whether a missed diagnosis of AF led to inappropriately withholding prescription of otherwise indicated OAC. We also collected data on the applied acute stroke treatment, including information if mechanical thrombectomy, i.e., the mechanical interventional procedure by which a blood clot or thrombus is removed under image guidance using endovascular devices, or intravenous thrombolysis was performed.

At the stroke units of Charité University Hospital Berlin, the standard operating procedures (SOP), that is herein assessed, stipulate a 12-channel ECG as one of the first diagnostic steps followed by continuous ECG monitoring at the stroke unit supported by an automated AF detection algorithm. Potential alarms are sent to the nurse's working spaces or the surveillance room. If a noticeable change in the ECG is spotted, one is required to rewind the recording for a more detailed evaluation. Additionally, once per day, the treating team prints out the automated alarms saved in the preceding 24 h, reviews and shelves them. In case of uncertainty a 12-lead ECG is recorded or a cardiologist is consulted. ECG monitoring is only interrupted for defined examinations or treatments. If the stroke is of potential cardiogenic cause, or when no other etiology is suspected, a long-term ECG for 24, 48, or 72 h, or recordings via ECG patch or loop recorder constitute additional workup options.

### 2.2 Validation of atrial fibrillation alarms, operating procedures

For patients in whom an AF alarm was recorded, we first downloaded ECG lead II data together with the respective alert announcement and alert onset timestamps for each AF alarm from the data lake. These timestamps correspond to the onset time of the AF pattern, as detected by the algorithm, and the time when the alarm is announced. The corresponding ECG data of each AF alarm was then bandpass filtered (phase-neutral filter, 0.5–40 Hz bandpass, sampled at 500 Hz) and plotted along with markers of the announce and onset times of each AF alarm. Fig. 3 provides an example of two alarms with the corresponding ECG trace and markers of alert onset and announce times. To be able to conclusively screen an alarm for AF we plotted and evaluated the ECG trace for 65 s for each alarm: from 5 s before the alert onset to 60 s thereafter. Each plot, corresponding to one alarm, was saved as an individual pdf-file. We restricted the number of ECG segments per patient to a maximum of the first ten alarms. Next, we uploaded these ECG visualizations into a dedicated REDCap (Research Electronic Data Capture) database [15,16]. Using this database, the ECG of each AF alarm was then categorized into three categories: AF, non-AF, or artifacts. Because of the similar clinical consequences, atrial flutter was subsumed as AF. The second category contained all non-AF and non-atrial flutter rhythms, including other cardiac arrhythmias or sinus rhythms. AF was defined as irregular RR intervals without clearly distinguishable and repeating P-waves and irregular atrial activity for at least 30 s [9]. The third category consisted of the ECGs being uninterpretable due to artifacts. Categorization was first performed by a physician (M.A.), if this physician was unsure about the category or a potentially missed true positive alarm was identified a board-certified cardiologist (A.B., E.M.) would double-check the reading and make a final decision.

After the evaluation of the alarms, classifications were matched with the medical history and discharge diagnoses as well as treatment strategy for each patient extracted from discharge letters. Specifically, we checked whether an AF diagnosis was missing on the discharge letter although at least one true positive alarm had been identified. Furthermore, in each of these patients, we checked whether a pharmaceutical AF treatment would have been indicated based on clinical guidelines. If the patient was more than once hospitalized due to an ischemic stroke episode during the defined time period, only data from the first stay was obtained.

### 2.3 Outcome measures

The main outcome was the number and proportion of AF diagnoses correctly identified and announced by the monitoring system but missed by the clinical team. Secondary outcomes included the proportion of those patients that triggered the missed correct alarms in whom a new diagnosis of AF would likely have led to the initiation of anticoagulant therapy. We also evaluated the performance of the automated detection system by determining the positive predictive value (PPV).

### 2.4 Statistical analysis

Missed AF diagnoses were counted in terms of number of patients and as a percentage of all patients included in the study without known AF before stroke. Missed anticoagulant therapy initializations were counted in terms of their total number and as a percentage of all patients in whom AF diagnosis was missed. The confidence interval was obtained by applying a Wilson Score Interval with continuity correction.

The performance of the automated detection system was evaluated by calculating the PPV, i.e., the number of true positive alarms divided by the number of all alarms. Its 95% confidence interval was calculated by bootstrapping (n = 200).

## 3 Results

### 3.1 Dataset

**Fig. 1:**
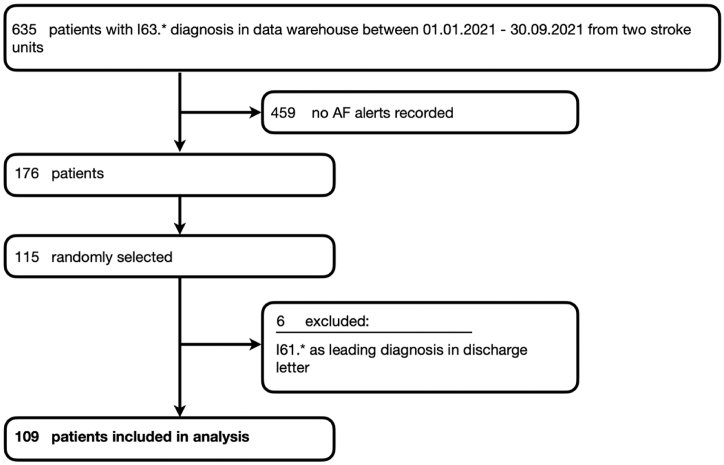
Flow-chart of patient selection.

**Fig. 2:**
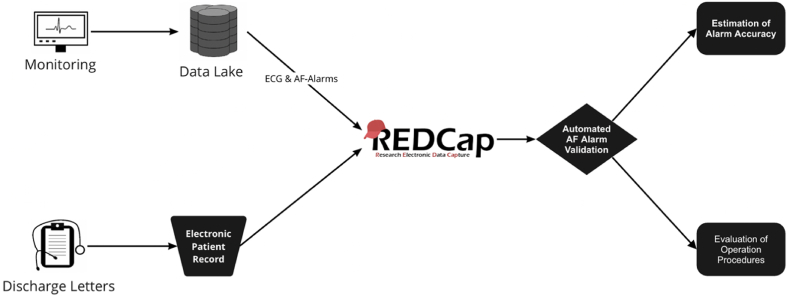
Data sources and data flow. Data stored in the data lake (ECG, diagnoses, patient characteristics such as age and gender, etc.) and information from discharge letters (including medication prescribed at discharge and NIHSS at discharge) were transferred to a REDCap database for evaluation.

635 patients with ischemic stroke diagnosis in a data warehouse were retrospectively identified when restricting the data to the time period between 01. January and 30. September 2021. Of those patients, 176 patients had recorded AF alarms, of which 115 patients were randomly selected for evaluation. This random selection was done to limit the time and workload required to fully evaluate the ECG segments and patient histories. 6 patients had to be excluded as their discharge letters – in contrast to the recordings in the data warehouse - revealed haemorrhagic stroke (I61*) as leading diagnosis. 109 patients remained for the final analysis (Fig. 1). Fig. 2 provides an overview of the data and data flow relevant to this study.

**Fig. 3:**
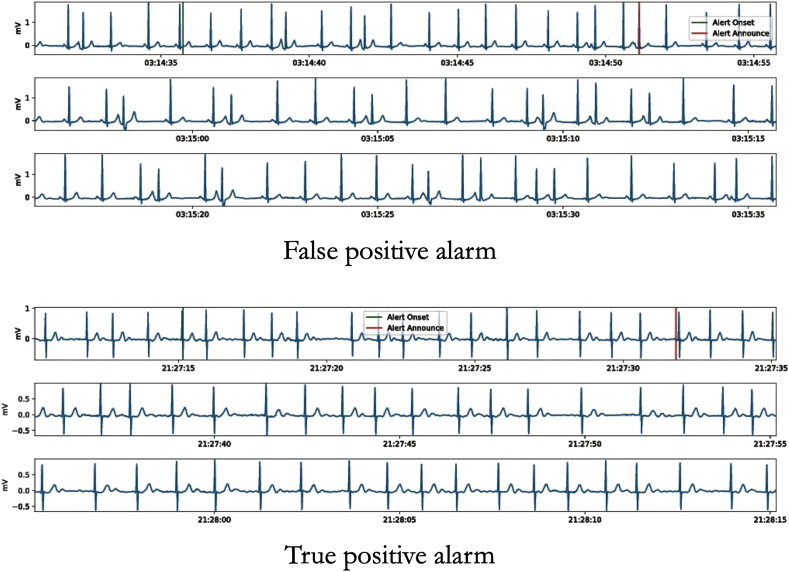
Examples of ECG data segments of false and true positive alarms. Top: example of a false positive alarm. Bottom: example of a true positive alarm. The green markers depict the arrhythmia onset, as detected by the algorithm. The red markers depict the alarm announcement times. (For interpretation of the references to colour in this figure legend, the reader is referred to the Web version of this article.)

With restriction to a maximum of ten alarms per patient, we obtained a dataset consisting of 717 automated alarms from 109 patients (mean: 6.6 alarms/patient). The ECG monitoring duration ranged from 2 to 38 days (median: 7 days, IQR: 5–11 days). At the time of admission to the stroke unit, the National Institutes of Health Stroke Scale (NIHSS) ranged from 0 to 23 (median: 6, IQR: 2–13). The main patient characteristics are summarized in Table 1. Out of 82 patients with an AF diagnosis at discharge, 34 (41.5%) had received solely mechanical thrombectomy, 6 (7.3%) only intravenous thrombolysis, and 9 (11%) both treatments. In terms of secondary prevention for 75 (91.5%) patients OAC (oral anticoagulants) and for 3 (3.7%) patients full-dose Heparin was prescribed. The most frequently recommended OAC was Apixaban (61 patients, 74%).

**Table 1:** Patient characteristics. ^1^The percentage value of anticoagulants recommended at discharge relates to the number of patients with diagnosed AF at discharge (82 patients).

	Absolute Values	Percentage % (95% CI)	Median (IQR)
**Numbers of patients included**	109		
Mean age (years)	79.1		80 (75–85)
Sex (female/male)	47/62	43/57	
AF diagnosis at admission	62	57	
NIHSS at admission	0–23		6 (2–13)
Duration of monitoring (days)	2–38		7 (5–11)
Mechanical Thrombectomy	50	46	
Thrombolysis	19	17	
**Total exitus letalis/Number of patients at discharge**	5/104	5/95	
AF diagnosis at discharge	82	75	
Missed AF diagnosis at discharge	4	3.7 (1.18–9.68)	
NIHSS at discharge	0–22		2 (0–5)
Anticoagulant at discharge	75	91^1^	
Apixaban	61	74^1^	
Dabigatran	7	9^1^	
Heparin	3	4^1^	
Rivaroxaban	3	4^1^	
Edoxaban	1	1^1^	

### 3.2 Alarm validation

**Fig. 4:**
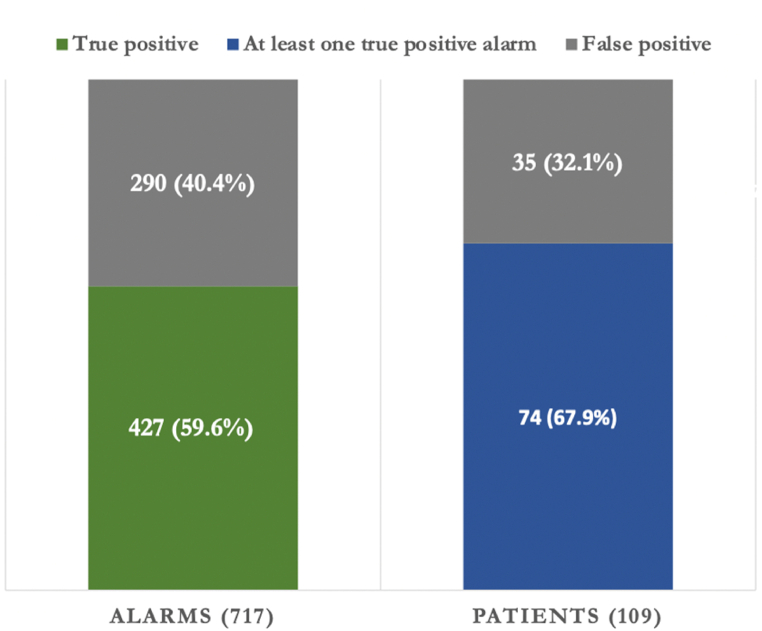
Alarm validation results. Left: 427 (59%) of the alarms were true positive, resulting in a PPV of 0.59. Right: 74 out of 109 patients (68%) had at least one true positive alarm. Between one and ten ECG segments were evaluated per patient.

Fig. 3 provides an example of two alarms with the corresponding ECG trace and markers of alert onset and announce times. 427 out of 717 alarms (59.6%) were rated as AF or atrial flutter during manual review and were thus considered true positives. From the remaining alarms 222 (31%) were counted as false positives, and 68 (9%) ECG traces showed strong artifacts. The resulting PPV amounted to 0.6 (95% CI 0.56–0.63). Out of 109 patients 74 (68%) had at least one true positive alarm and 35 (32%) had none (Fig. 4).

### 3.3 Evaluation of the AF workup

**Fig. 5:**
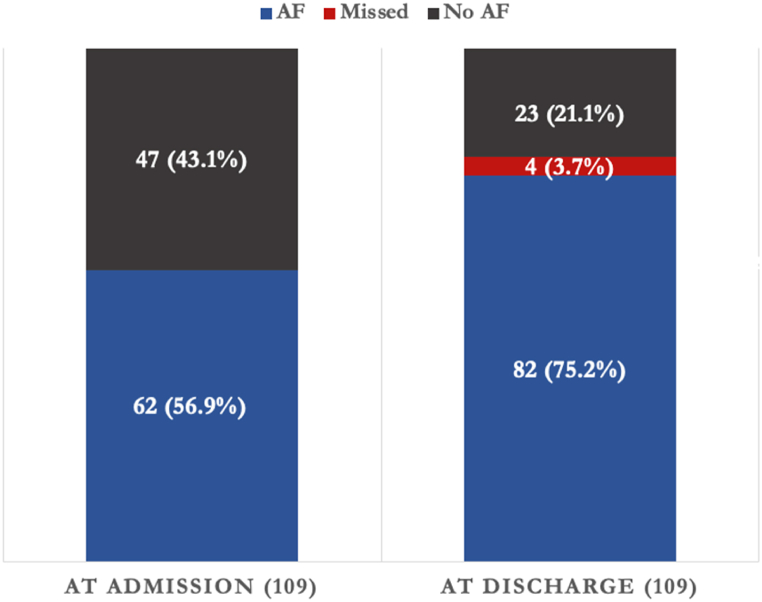
Results of operating procedure assessments. Left: In 62 (57%) patients, AF was known before clinical monitoring. Right: During hospitalization AF was identified in 20 (18%) additional patients. Four patients should have been diagnosed when considering the missed true positive AF alarms.

At admission, 62 patients (56.9%) had already been diagnosed with AF (Fig. 5). Until discharge, an additional 20 patients received an AF diagnosis, resulting in a total of 82 patients (75%). In four patients a valid true positive AF alarm had remained undetected, so that in 3.7% (95% CI 1.18–9.68) of all included patients and in 8.5% (95% CI 2.76–21.27) of all included patients without a prior known diagnosis of AF the treating physicians had missed the AF diagnosis. All four patients did not receive OAC as long-term secondary prevention at discharge (Fig. 5). In two of these patients information in the letter of discharge pointed to potential contraindications for anticoagulation. Specifically, both were affected by relevant unsteady gait. One additionally showed cerebral microbleeds (CMB), probably due to hypertension, in the MRI when examined during the hospitalization and was suspected to have Cerebral Amyloid Angiopathy (CAA). For the two remaining patient no apparent contraindications for OAC were found in their discharge letter. Sadly, for one patient without a contraindication a recurrent ischemic stroke was documented in the electronic health records eight months after the first one.

## 4 Discussion

In everyday clinical workup, standardized procedures are crucial for an efficient workflow. Standards of procedures may vary between centers, sometimes even within a single hospital. With the advent of automated technologies, including automated alarms for AF detections, regular checks of error rate and accuracy of both the standard of procedures and the performance of automated alarms are important yet also resource-intensive tasks. With the increasing availability of data warehouses, new opportunities arise for validation of these procedures and alarms. In this work, we present an integrative pathway for this purpose.

The establishment of data warehouses for comprehensive and long-term data storage may afford more effective and reproducible evaluation of alarms and operating procedures. Once a data quality control pipeline is set up, such as in the present study, the process may easily be replicated or revised. This may help to uncover potential factors influencing performance, such as changes in a unit's personnel or the standard of procedures entailing quality control. The assessment of a single patient's data warehouse-stored AF alerts - like the workup in this study - could also be considered a regular task each time a patient is discharged. This real-time quality control may potentially facilitate direct feedback to the treating physicians and nurses and afford timely rectification of the treatment plan in case of missed alarms, for example.

We consider the rate of 3.7% of missed AF diagnoses observed here due to inconsistent checking of all automated alarms to be low but improvable. Sposato et al. suggest that a substantial part of AF is still not detected at discharge. This meta-analysis showed that in at least 11% of the patients, AF was detected by monitoring with ambulatory Holter, outpatient telemetry, and external or implantable loop recording thereafter [3], although the applied definitions of AF diagnosing differed between the included studies. Future investigations could try to identify the underlying reasons which lead to our result, e.g., fluctuations of personnel or the influence of alarm fatigue. However, based on the low number of missed alarms observed in this study, it may require analyses of larger patient cohorts.

All four patients with a missed AF diagnosis did not receive secondary prevention at discharge. However, in two of these three patients, conditions were reported that may be considered contraindications to anticoagulation. The benefit of OAC may be questioned and some physicians may be reluctant to prescribe OAC under those conditions [17,18].

More generally, the performance of alarms used in every-day clinical workups, such as the AF alarm studied here which forms a center pillar determining treatment decisions in stroke patients, are often not fully known or have been only insufficiently validated. Clinical data warehouses are highly suitable to address such important tasks. They allow semiautomated investigations of substantial amounts of data and may help to make even smaller influences on treatment quality more measurable. Other metrics, such as sensitivity or specificity may be interesting metrics of investigations in future studies. With the advent of more automated and artificial intelligence algorithms used during patient work-up, it will also be important to determine the accuracy and validity of these algorithms in routine clinical settings. Quality control pipelines built on data warehouse structures provide an ideal setting for this. Such a pipeline may allow for the selection of the best-performing algorithm for a specific clinical setting. D'Anna et al. compared two cohorts, one admitted to continuous stroke unit ECG monitoring (CEM) and the routine clinical practice which also deployed Philips monitors versus another group undergoing additional screening with software (SRA) for automated AF detection followed by another review of the alarms by a specialist [19]. In the second group, there were 5.7% points more AF detected. Another study found a 2.3% better detection of automated CEM also using SRA software [20]. These findings suggest that using the Philips AF detection algorithm for quality control could lead to an underestimation of missed AF diagnoses in comparison to other technologies with higher sensitivity. Here, we only focused on detection PVV, but incorporating a sensitivity analysis is in principle also feasible with the proposed analysis pipeline. Better insights into these alarms and their iterative optimization may also help to reduce alarm fatigue [1]. Intense alerting due to too low specificity in an intensive care unit has been reported to be associated with decreased productivity levels of nurses and reduced trustworthiness into the alarms [21] with the consequence that alarms may get ignored at times. While the benefits of automated AF alarms are undisputed, the ideal setting and procedure for effective review in clinical practice must be specified. This is embedded in the question of the most effective strategy to detect AF in general [22].

A limitation of this study concerns the quality of the ECG data. The sequences are automatically stored regardless of potential movement artifacts, which are unavoidable when the patient is monitored without interruption. Therefore, validation with only one lead was sometimes challenging and, in some cases, not possible at all. Studies using a data warehouse structure are dependent on its maintenance and data quality. Data warehouse data is often not as curated and “clean” as the data from highly curated clinical studies. However, it is often orders of magnitudes larger in size and may more directly reflect “real-world” conditions. We here did our best to clean the data and have it evaluated by experts. Also, we omitted to further investigate the underlying causes leading to undetected alarms, which may include staff composition and experience, work shift dependencies or patient specific factors. As discussed, clinical data warehouses may provide an ideal basis for this task and will be the focus of future research. Further, the effect of refrained anticoagulant therapy on the outcome in patients with missing AF diagnosis is yet unclear. This follow-up may be addressed by future prospective studies. Finally, it is important to note that results of this study are limited to the two stroke units and may not generalize to other tertiary care hospitals. The performance evaluation here was applied to one specific monitoring system and other monitoring systems could be more efficient in the detection of AF and signaling it.

## 5 Conclusion

In summary, the implemented SOPs to detect AF using automated AF detection appeared suitable, documenting only few missed AF diagnoses. This relatively high accuracy came at the cost of false positives, resulting in a positive predictive value of 0.6. A data warehouse structure storing ECG data-related alarms, electronic patient records and a central survey tool, such as REDCap enables data quality monitoring. Such pipelines may help to adjust internal processes or identify targets for further investigation into and adaptation of operating procedures.

## Data availability statement

Data are not publicly available due to the data protection status.

## Patient consent for publication

Not applicable.

## Author contribution statement

Mario E. Andina, Alexander Nelde, Eckhard Meisel, Anne Bingel: Performed the experiments; Contributed reagents, materials, analysis tools or data; Wrote the paper.

Christian H. Nolte, Jan F. Scheitz, Manuel C. Olma, Michael Krämer, Andreas Meisel, Franziska Scheibe, Matthias Endres: Contributed reagents, materials, analysis tools or data; Wrote the paper.

Ludwig Schlemm, Christian Meisel: Conceived and designed the experiments; Contributed reagents, materials, analysis tools or data; Wrote the paper.

## Declaration of competing interest

CM is part of patent applications to detect and predict clinical outcomes and to manage, diagnose, and treat neurological conditions, all of which are outside the submitted work. CHN received research grants from German Ministry of Research and Education, German Center for Neurodegenerative Diseases, German Center for cardiovascular Research, and speaker and/or consultation fees from Abbott, Alexion, Astra Zeneca, Bayer Pharma, Bristol-Myers Squibb, Daiichi Sankyo, Novartis, Pfizer Pharma, Portola and Takeda all outside the submitted work. ME reports grants from Bayer and fees paid to the Charité from Abbott, Amgen, AstraZeneca, Bayer, Boehringer Ingelheim, BMS, Daiichi Sankyo, GSK, Sanofi, Covidien, Novartis, Pfizer, all outside the submitted work. The remaining authors have no conflicts of interest or competing interests to declare.

## References

[1] Ruppel H., Funk M., Whittemore R. (2018). Measurement of physiological monitor alarm accuracy and clinical relevance in intensive care units. Am. J. Crit. Care.

[2] Saposnik G., Gladstone D., Raptis R. (2013). Atrial fibrillation in ischemic stroke. Stroke.

[3] Sposato L.A., Cipriano L.E., Saposnik G. (2015). Diagnosis of atrial fibrillation after stroke and transient ischaemic attack: a systematic review and meta-analysis. Lancet Neurol..

[4] Borowsky L.H., Regan S., Chang Y. (2017). First diagnosis of atrial fibrillation at the time of stroke. Cerebrovasc. Dis..

[5] Zhang C., Kasner S.E. (2015). Paroxysmal atrial fibrillation in cryptogenic stroke: an overlooked explanation?. Curr. Atherosclerosis Rep..

[6] Hart R.G., Pearce L.A., Aguilar M.I. (2007). Meta-analysis: antithrombotic therapy to prevent stroke in patients who have nonvalvular atrial fibrillation. Ann. Intern. Med..

[7] Ruff C.T., Giugliano R.P., Braunwald E. (2014). Comparison of the efficacy and safety of new oral anticoagulants with warfarin in patients with atrial fibrillation: a meta-analysis of randomised trials. Lancet Lond. Eng..

[8] Lip G.Y.H., Keshishian A., Li X. (2018). Effectiveness and safety of oral anticoagulants among nonvalvular atrial fibrillation patients. Stroke.

[9] Hindricks G., Potpara T., Dagres N. (2020). ESC Guidelines for the diagnosis and management of atrial fibrillation developed in collaboration with the European Association for Cardio-Thoracic Surgery (EACTS): the Task Force for the diagnosis and management of atrial fibrillation of the European Society of Cardiology (ESC) Developed with the special contribution of the European Heart Rhythm Association (EHRA) of the ESC. Eur. Heart J..

[10] Gladstone D.J., Spring M., Dorian P. (2014). Atrial fibrillation in patients with cryptogenic stroke. N. Engl. J. Med..

[11] Powers W.J., Rabinstein A.A., Ackerson T. (2019). Guidelines for the early management of patients with acute ischemic stroke: 2019 update to the 2018 guidelines for the early management of acute ischemic stroke: a guideline for healthcare professionals from the American heart association/American stroke association. Stroke.

[12] Kleindorfer D.O., Towfighi A., Chaturvedi S. (2021). Guideline for the prevention of stroke in patients with stroke and transient ischemic attack: a guideline from the American heart association/American stroke association. Stroke.

[13] Marta Rubiera, Ana Aires, Antonenko Kateryna, Lémeret Sabrina, Nolte Christian H., Putaala Jukka, Schnabel Renate B., Tuladhar Anil M., Werring David J., Zeraatkar Dena, Paciaroni Maurizio (2022). https://journals.sagepub.com/doi/full/10.1177/23969873221099478.

[14] Koennecke H.-C., Belz W., Berfelde D. (2011). Factors influencing in-hospital mortality and morbidity in patients treated on a stroke unit. Neurology.

[15] Harris P.A., Taylor R., Thielke R. (2009). Research electronic data capture (REDCap)—a metadata-driven methodology and workflow process for providing translational research informatics support. J. Biomed. Inf..

[16] Harris P.A., Taylor R., Minor B.L. (2019). The REDCap consortium: building an international community of software platform partners. J. Biomed. Inf..

[17] Charidimou A., Shoamanesh A., Al-Shahi Salman R. (2018). Cerebral amyloid angiopathy, cerebral microbleeds and implications for anticoagulation decisions: the need for a balanced approach. Int. J. Stroke Off. J. Int. Stroke Soc..

[18] Badi M.K., Vilanilam G.K., Gupta V. (2019). Pharmacotherapy for patients with atrial fibrillation and cerebral microbleeds. J. Stroke Cerebrovasc. Dis. Off. J. Nat. Stroke Assoc..

[19] D'Anna L., Kar A., Brown Z. (2020). Automated continuous electrocardiogram monitoring accelerates the detection of atrial fibrillation after ischemic stroke or transient ischemic attack on a hyper acute stroke unit. J. Stroke Cerebrovasc. Dis..

[20] Rizos T., Güntner J., Jenetzky E. (2012). Continuous stroke unit electrocardiographic monitoring versus 24-hour holter electrocardiography for detection of paroxysmal atrial fibrillation after stroke. Stroke.

[21] Gomis M., Dávalos A., Purroy F. (2020). Stroke risk analysis, a system with a high detection rate of atrial fibrillation in stroke and transient ischemic attack. Stroke.

[22] Cuadrado‐Godia E., Benito B., Ois A. (2020). Ultra‐early continuous cardiac monitoring improves atrial fibrillation detection and prognosis of patients with cryptogenic stroke. Eur. J. Neurol..

